# Digital intervention for public health: searching for implementing characteristics, concepts and recommendations: scoping review

**DOI:** 10.3389/fpubh.2023.1142443

**Published:** 2023-09-18

**Authors:** Hatem H. Alsaqqa, Abdallah Alwawi

**Affiliations:** ^1^Deanship of Scientific Research, Al-Quds University, Jerusalem, Palestine; ^2^Ministry of Health, Gaza Strip, Palestine; ^3^Anesthesia and Resuscitation Technology, Health Professions Faculty, Al Quds University, Jerusalem, Palestine

**Keywords:** characteristics, concepts, digital interventions, implementation, public health

## Abstract

Studying the impact of digital interventions on public health can help ensure that the offered services produce the desired results. In order to address these factors, the subsequent study uses a scope review to evaluate the state of the field while concentrating on ideas and suggestions that represent factors that have been crucial in the management of digital intervention for public health. To shed light on the traits, ideas and suggestions related to public health digital intervention, a scoping review was carried out. Five electronic databases were used to locate pertinent research that were published before February 2022. All texts were examined, and study abstracts were scrutinized to determine their eligibility. The last analysis of this study included fifteen publications; five reviews, four qualitative studies, two quantitative studies, one viewpoint study, one mixed-method study, one perspective study, and one interventional study. The key ideas for digital interventions in population management and health studies are presented in this overview. Many concepts, implementation characteristics and recommendations have been raised which highlight the future role of these interventions to enhance public engagement and health equity.

## Introduction

The choice of an appropriate theory to administrator the implementation course and the technique selection, assuring that proper consideration is paid to planning implementation, and having a flexible tactic that allows for response to recently evolving obstacles are all of the utmost importance.

Public health professionals and academics have proposed a variety of responses in their search for solutions, including stepping up current initiatives to promote information and health literacy, coming up with plans for widely refuting distortion, and educating clinicians and public health professionals on how to discourse misinformation one-on-one (1).

Better data science ought to lead to healthier behaviors and wiser health decisions. In this way, technologically mediated health data processing might support patient empowerment and individual sovereignty (2, 3). However, because human decision-making is complex and influenced by environment and intellectual biases, mixing emotion and rational, the embracing of healthy habits does not occur linearly as a result of better health knowledge. In addition to being the area of health care that is focused with promoting healthy behavior, health promotion is viewed as crucial to attempts to avoid diseases (4).

However, methods from all these fields are needed since these studies sit at the nexus of biological, behavioral, computational, and engineering research. Related research answers encompass identifying the issue and the expected assistance of the digital health intervention (DHI), which in flip necessitates determining the intervention’s likely reach and uptake, the causal model outlining how the intervention will produce the optimally selected, key elements and how they socialize with one another, and assessing the actual advantage in terms of effectiveness, cost effectiveness, and harms (5).

Distinguishing the implication of digital technology in this expanse and in pandemic preparation planning has become crucial since the future of public health is anticipated to be more and more digital. Technology productions and other noteworthy players in the digital area ought to work together as longstanding allies in readiness rather than only through crises times (6).

DHIs, which are therapies given through digital technology like smartphones or websites, have a huge potential to provide efficient, affordable, safe, and scaled interventions to promote healthcare. DHIs can be used to optimize outcomes for those with longstanding conditions, such as cardiovascular disease, diabetes, and mental health issues, as well as to provide remote access to effective treatments. They are frequently intricate interventions with numerous parts, and many of them have multiple goals, such as empowering users to learn more about their health, connect with others in a similar situation, alter perceptions and beliefs about it, monitor certain health conditions or behaviors, titrate prescription, identify treatment priorities, and enhance patient-provider interactions (5).

This review aims to examine the series of digital inventions used universally to address public health challenges, as well as their restrictions and implementation footraces, such as those associated to the law, ethics, and privacy as well as legislative and personnel issues. The objective of the paper is to identify mechanism-based explanations for how and in what contexts digital intervention for public health achieved its effects.

## Appraising digital health interventions

Integrating assessment from the start of the DHI progress process allows for the development of evaluation thinking, abilities, and tools. The resulting evaluation service gives non-academics and digital developers the ability to use evaluation approaches and thinking when designing, developing, and implementing their DHI. By doing this, it demystifies evaluation, which has historically been the purview of academia, and uses people’s motivations to make sure that their DHI is as effective as it can be while enhancing the health and wellbeing of end users (7).

Diagnostic or population health interventions, digital product design, product and service design, as well as communication and health promotion, are all components of the interdisciplinary endeavor known as DHIs. Therefore, interdisciplinary approaches to evaluation are best for understanding the effectiveness of DHIs as well as their usableness and attractiveness, with success criteria that consider the various parties involved in the hiring, design, and growth of a DHI as well as its end users (8). To validate the appraisal enterprise path for a DHI, seven key ideas for evaluating DHIs have been identified; evaluation thought, review image, contract assistant, testing tools, progress history, data hub, and published health results.

### Argue the structural and epistemic aspects

Along with the crucial problems of safety, data privacy, and the value of human caring touch, structural unfairness raises concerns. DHIs for “reporting and evidence building” urge users to actively recount their involvements and join with other survivors’ tales, creating a shared epistemic space for people who have come into contact with violence. Users are encouraged to apply their epistemic capacity, are recognized as epistemic subjects, and are able to communicate and possibly advance their knowledge within the user community by allowing consumers to express their opinions, even if only digitally through a digital application.

These DHIs can thus stress the need to respond not only on an interpersonal basis but also on a structural level and may aid in better comprehending patterns and clusters of violence (e.g., societies, regimes). These DHIs might also make people impress less alone in their involvements, teach them coping mechanisms, and help them locate guidance and support. Even if applications are created in a way that considers and reflects systemic factors of violence, their impact would be dubious if not everyone can use them. By endorsing digital intervention tactics that can only be opened by users with specific advantages, this runs the risk of highlighting and strengthening structural and epistemic unfairness.

The main epistemic circumstances and traits are recognized under the categorization: data and information structures are related to psychological effects in four ways: (1) they are caused by psychological properties, (2) they are caused by information features, (3) they are related to psychological properties, and (4) information features (co-)constitute psychological properties (9).

Additionally, as DHIs become more prevalent as an intervention strategy, problems like the loss of private contact in intervention settings (such as social workforces providing resident counseling) as a result of a change to digital technology, the possibilities for mistranslation of the details given owed to the absence of non-verbal cues, as well as matters with language and comprehension and access to technology, may crop up (10).

Meanwhile, social media usage for purposes related to health has the ability to create interpersonal networks that support specific epistemic positions on medical matters, which could have a negative impact on public health. Another illustration is the impact of technology-mediated interaction on the connection between the patient and the healthcare professional (11).

### Personal agency and motivation

As patients and the general public tended to engross with and enroll in DHIs because they wanted to be healthy or have more influence over how they managed their welfare, the first topic that arose was personal agency and motivation. Information technology was believed that using technology could help people stay motivated to engage in physical activity, reduce their weight, and stay healthy (12, 13). As a result of having the freedom to obtain health information whenever and wherever they pleased, many people joined a DHI, which in some circumstances helped lower anxiety (14, 15). The level of regulating knowledge provided for tracking and comprehending health-related behaviors, such as food and exercise, or for managing chronic diseases on one’s own, was also well-liked by users, which prompted registration (12).

### Personal life and values

The recurring topic was how patients’ and the general public’s capacity to participate in and participate in DHIs was impacted by a busy personal life with many conflicting demands. People tended to sign up for new technology if they felt it was useful, could be customized to meet their needs, and was simple to integrate into their daily lives (15, 16). Additionally, individuals who were digitally literate (14, 16) and had experience with or were already familiar with utilizing technology (14) found it simpler to enroll since they possessed the necessary knowledge and abilities. Some people registered because they valued the privacy that online health services offered, being secure and protected from the discrimination and disgrace they occasionally encountered in the actual life (12, 16).

## Perceived fit perceived

In contrast to a one-size-fits-all approach, perceived fit describes how much users handled the intervention was acceptable, applicable to their culture and values, and/or oriented at others who were similar to them. For instance, the information’s applicability to their current circumstance (17, 18) and the ability to adapt or tailor the intervention (19, 20) made it more likely that it would fit. Users’ ability to relate to the intervention’s presenters, who may be coaches, teachers, or samples of people with comparable situations, was a facilitating element (21). Culturally appropriate material (22), level of literacy (23), and content given with little use of technical terminology (24) are all examples of elements that make the information pertinent and in a vocabulary suited for the user.

## Perceived usefulness

The term “perceived usefulness” describes how a user feels about an intervention and how they judge if it will be helpful to them. Users’ ability to comprehend the information presented to them (104,117,170), the clarity of the action they should take (17, 25), and the perception that the intervention offered a distinct advantage over previous or ongoing care received (17, 26) all contributed to this perception. Facilitators were identified as making it simpler for users to get services they would not otherwise have access to (26, 27) and removing the need for them to travel far to a health center (28).

## Level of guidance

The amount of assistance a user receives when using an intervention—for instance, through reminders or a web-based supporter—determines how much accountability they receive to frequently engage with the information. If the intervention raised the level of control, leading to users perceived more responsibility over their own health, it would be a facilitating factor for utilizing DHIs (29, 30). Participants had trouble interacting with interventions that were entirely self-guided, and they occasionally failed to use the intervention (31). The demand for more structured use was voiced by the participants, for examples of this structured use include app alerts or routine human coaching checks (32, 33).

## Social connectedness

User engagement was revealed to be facilitated by an intervention’s impact on participants’ feelings of social connectivity. Another facilitating element was if the intervention (34, 35) helped to mainstream lived perspectives by giving instances of others who had comparable experiences. Additional beneficial outcomes that could promote engagement included enhanced abilities (36, 37), a greater understanding of users’ health (38, 39), and a sense of empowerment over having control over their wellness (40).

### Iterative methods to adjust an intervention

Regular stakeholder involvement, new scientific information, evolving government directives, quick qualitative research (telephone interviews and open-text questionnaires), and usage data analysis all influenced the optimizations. All comments were quickly compiled, and potential improvements were prioritized according to their likelihood of having an influence on behavior change (41).

In order to improve a health intervention and/or its execution to achieve stakeholder-specified public health benefits within supply limits, optimization can be defined as a purposeful, iterative, and data-driven procedure. This study sought to characterize the core ideas, procedures, or processes of selected frameworks to maximize the efficacy of health interventions and/or their administration (42).

The optimization step’s goal was to test and perfect the intervention’s reasoning and program theory in order to comprehend intervention mechanisms and increase its effectiveness. This frequently happened by doing numerous or repeated “little experiments.” If the “experimentation” step was unsuccessful, the intervention may then go back to the preparation or “theoretical/literature base” phase (42).

An intentional, iterative, and data-driven effort to enhance a health intervention is what is meant by optimization (43). In-depth qualitative research pinpoints obstacles to behavior and intervention adoption and iteratively improves the intervention to get around them (44, 45). The theory- and evidence-based behavioral analysis is integrated into this strategy to choose the most suitable set of efficient behavior change strategies (46).

A live intervention’s effectiveness was improved quickly and iteratively to keep pace with the terrifying and continuously changing environment of an international crisis. Making sure the intervention’s contents is inspiring, credible, and convincing may be more crucial for fostering involvement than making changes to the intervention’s design (41).

### Key principles of intervention development

Important guidelines for developing interventions should follow certain key tenets, including being dynamic, iterative, innovative, adaptable, and forward-looking in terms of evaluation and application in the future. When developing an intervention, developers are likely to switch back and forth between redundant tasks including examining the available data, using preexisting theory, and interacting with stakeholders. Iterative cycles will also be used to establish a rendition of the intervention, with feedback from stakeholders used to identify issues, possible alternatives put into action, their appropriateness evaluated, and the cycle repeated until evaluation of subsequent iterations of the intervention shows rare variations.

The need for the intervention, its style, substance, or method of delivery may be strongly held beliefs when the intervention is first being developed. Throughout the design process, keeping open to various alternatives may result in abandoning the project or moving both backward and forward. Being adaptable is a good idea since it could lessen the likelihood that you produce an intervention that fails in a later evaluation or is never used in practice. In order to prepare for this and highlight lessons learned and significant uncertainties that need to be addressed in future evaluations, developers may also gain from anticipating how the intervention will be appraised (47).

Monitoring and iterative evaluation should be prioritized to the greatest extent possible, and results should be regularly discussed and understood in collaboration with stakeholders, as well as thoughtfully and continually implemented in any system redesign or anticipated adaptations/modifications (48). The iterative method of data collection, assessment, evaluation, review, and change responds to the dynamic nature of evidence and the requirement for learning from and with stakeholders, such as populations and field workers (49).

## Patient and public involvement

By raising disease understanding and recognizing patients as active participants in their own conditions, patient and public involvement (PPI) can support patient empowerment (50). However, PPI varies significantly between nations and research organizations, and even today, many patients and the general public do not participate in or have access to study protocols (51). Cohort studies are increasingly including digital resources like websites, social media, and connected gadgets, which could be used to boost PPI (52). Digital tools can also help PPI by facilitating feedback and communication between study collaborators and patients (53). PPI is an effective strategy for raising the relevance of research efforts. We have demonstrated that PPI must be designed from the early stages of the construction of a original epidemiological study and then deliberated as the research project progresses. The most successful technique for raising the caliber of research appears to be combining various PPI approaches (54).

## Contextual indicators

The contextual indicators basis was created to offer direction on the interdependent context, implementation, and setting characteristics that may have an impact on the efficient delivery of complex interventions (55). What is crucial is that the framework explains how ambitious contextual factors outside of the administrative environment may affect how a complicated intervention with community-facing components is implemented. The seven dimensions that contextual indicators consider are; geographical, epidemiological, sociocultural, socioeconomic, ethical, legal, and political setting. Here, researchers look into the interactions between the political (healthcare infrastructure), epidemiological (blood pressure, body mass index, and older population) and geographical (region, urbanicity) domains of the contextual indicators (56).

### Share viewpoints and knowledge from public health experts

The deeper understandings into participants’ involvements, opinions, views, and tips helped the researchers produce more detailed data and also helped others. Focus group involvement provided community members with new perspectives on issues they were discussing as well as a sense of insertion and community development, according to their reports (57).

Future studies should concentrate on a few unanswered problems about the use of digital forms in community-based health promotion interventions. If digital forms potentially take the place of outdated setups for health promotion and prevention actions, notably in vulnerable groups, this should be seriously evaluated. One of the most pressing unknowns, in our opinion, is whether the use of digital health promotion interventions results in an extra enlargement of a selection bias or whether such interventions combat this bias and are utilized and recognized by vulnerable groups and environments where inclusion struggle (58).

The researchers came to the conclusion that emphasizing participation in DHIs and utilizing standardized metrics to describe DHIs will aid future research and potentially open up more possibilities for meta-analyses of DHI results. This is further confirmed by Zanaboni et al. (59), who state that more emphasis should be given to clinical research in the form of high-quality randomized controlled trials in order to run a credible evidence base about the use of digital health and health results. According to Blandford et al. (8), established health research methodologies need to be flexible and modified in order to evaluate DHIs in study.

## Methodology

By using scoping review approach, this study investigates DHIs for public health. A review of the literature known as a “scoping study” or “scoping review” has the goal of “fastly mapping the major concepts driving a research topic and the main sources and forms of evidence available, notably where a subject is intricate or has not been studied thoroughly before” (60).

This kind of scoping review may not go into individual study findings, but rather maps and visualizes the body of knowledge that exists within the confines of the research field (61). Data were gathered and evaluated throughout five stages, as per the scoping review process delineated by Arksey and O’Malley (61), which is described below.

According to the stated rules for writing systematic reviews, peer reviews, and research articles, a systematic review was planned and carried out. The literature on digital interventions for public health has undergone a thorough assessment. The articles’ quality was not evaluated because it is not a part of the typical scoping review technique.

The main review interrogation was; “what are the implementing characteristics, concepts and recommendations of the digital interventions for public health considered.” In addition to updates in five databases, an electronic search of digital interventions for public health was conducted. We searched databases from EBSCO, PubMed, ScienceDirect, Scopus and the Cochrane Library. We looked for the words “digital intervention” and “public health” in the article titles. In relation to the objective of the study, it was determined that these terms should be used the most. Duplicate articles were removed, and articles had to have been released in English before February 2022. All scholarly investigations underwent a thorough search of the peer-reviewed literature.

**Figure 1:**
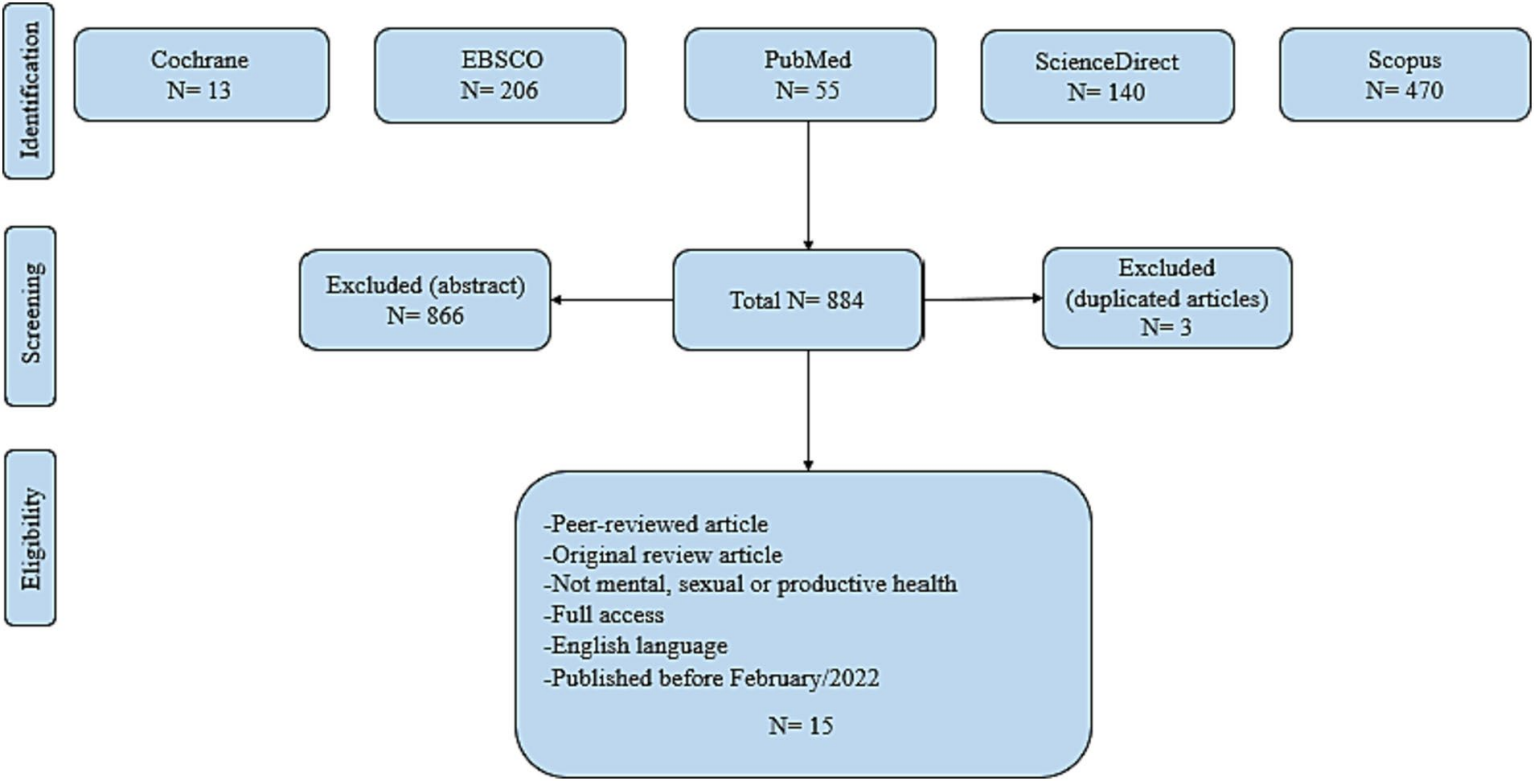
PRISMA flow chart of the literature review search.

Four main inclusion criteria were defined (Figure 1):

- Published papers as peer-reviewed.
- Original research articles.
- Papers with full access possibility.
- Not targeted mental, sexual or productive health research.
- Papers written in the English language.
- Published before February/2022.

Studies that did not match the aforementioned requirements were excluded, while those that did were listed and subjected to further evaluation. Studies were assessed and given a critical review. Extraction of the key conclusions from each repossessed study and literature screening (a three-stage technique involving exclusion by reading the title, the abstract, and the full text). The following details were taken from each of the studies that were included (Table 1): title, authors, country, study design, research objective, and key findings.

**Table 1:** A summary of reviewed studies.

Author	Setting/design	Aim	Study focus and findings	Recommendation
Burke and Bloss (62)	United States viewpoint	To hire commercial corporations to adopt cutting-edge technologies that monitor and manipulate students’ social media activities	There is a need for creative answers to the problems of student health and safety. The assertions made by social media monitoring firms and the schools that employ them that these technologies can solve the wide range of public health issues affecting pupils are unsupported by any evidence	The issues that young individuals already confront, particularly those from historically oppressed groups, may only be made worse by these digital surveillance tools
Susser (63)	United States perspective	Various strategies have been proposed by officials and public health experts, including; stepping up current initiatives to advance information and health literacy, coming up with plans for publicly refuting falsehoods, and training clinicians and public health authorities to deal with falsehoods one-on-one	Dealing with ethical challenges successfully will require balancing difficult tradeoffs. The vast amounts of personal information that have been gathered about each of us are tremendously illuminating, and the instruments for using that information to target digital communications are strong and easily accessible. It is simple to comprehend why academics and professionals in public health are keen to investigate the potential good they may do with them. The ethical costs of targeted digital public health initiatives may be high	Targeting technologies frequently infringe the security of personal information by using data that has been collected in this way. With these technology, disadvantaged populations could be targeted with discriminating messages. Targeted digital public health interventions pose a risk of interfering with our autonomy by influencing our decision-making. Each situation should be evaluated individually to determine whether the advantages of these interventions exceed the disadvantages. Practitioners should weigh the seriousness of the health problems they are addressing as well as their ability to reduce potential effects when making these decisions. It proposes a starting point for conversations on the morality of targeted digital public health interventions
Karpathakis et al. (7)	London multidisciplinary project team user-centered qualitative research	A framework for evaluation that integrates biological and digital methods was intended to be operationalized as part of the Public Health England (PHE) effort. Shows how effective, affordable, and beneficial DHIs are for improving public health	Seven key ideas for evaluating DHIs have been identified: evaluation thinking, evaluation canvas, contract assistant, testing tools, development history, data hub, and publish health outcomes. The planned PHE evaluation service for public health DHIs was developed after additional testing and refinement of three concepts that were given priority	PHE was able to integrate the skills of academic and biomedical fields with the knowledge of non-academic and digital developers through the use of an iterative, user-centered design methodology. Using design-led techniques in public health settings can be beneficial. The following service is now offered by health organizations in the UK and is called evaluating digital health products
Sauerborn et al. (10)	Germany evaluation content and functions apps	Constructed on an awareness of structural, societal, and individual dimensions of violence against women and girls as a multidimensional, global public health concern, and positioning it within the theoretical framework of structural injustice	Make the case that while technical tools like apps may be helpful in the battle against violence against women and girls, they must be positioned within the larger context of public health that takes into account the structural aspects of such violence. Along with major considerations for safety, data privacy, the value of human supportive touch, and other issues, structural injustice concerns are significant features in the ethical evaluation of such apps	Research on the function and applicability of apps as tactics to deal with the structural and epistemic aspects of violence is still lacking
O’Connor et al. (64)	UK systematic review	In order to guide future implementation efforts, it is important to identify and integrate the qualitative research on the factors that influence recruitment and involvement in DHIs	Four major topics that influence the participation of patients and the general public in DHIs emerged: (1) Individual agency and motivation; (2) the individual’s life and values; (3) the recruitment and engagement strategy; and (4) the DHI’s level of quality. Outlines the recruitment and engagement techniques used. To highlight the crucial steps, a draft digital health engagement model (DIEGO) was created. Future research recommendations are created after identifying existing knowledge gaps	Summarizes and elucidates the complexities of the recruitment and participation processes in digital health, as well as the problems that must be resolved before patients and the general public commit to digital health. It will take more effort to develop personalized, higher-quality digital solutions that are clinically accredited and endorsed when necessary. Additionally, more money is required to boost computer literacy and make sure that technologies are available and cheap for individuals who want to subscribe to them
Holst et al. (65)	Norway mixed methods (nonrandomized controlled trial and qualitative interviews)	To evaluate the DHI’s impact on rural communities’ long-term acquisition and retention of health knowledge	(1) Compare the intervention group’s knowledge ratings at baseline and immediately following the intervention. (2) The baseline knowledge score disparity between the intervention and control groups	Analyzing a DHI’s results in light of pertinent health messages
Budd et al. (66)	United Kingdom review	To document the range of technological developments for the global public health response to COVID-19 and its shortcomings	To identify obstacles to its execution, such as those posed by the law, morality, and privacy concerns, as well as those posed by organizations and the workforce	Examine the necessity of coordinating global strategies for the control, assessment, and application of digital technology to improve pandemic readiness and future COVID-19 and other infectious disease preparedness
Morton et al.(41)	United Kingdom review	Offers a collection of iterative techniques for quickly modifying and improving an intervention as it is being implemented	The intervention was clinically correct thanks to tight collaboration with clinical stakeholders. Contributors to patient and public involvement (PPI) recognized critical clarifications to the intervention’s content and made sure that data concerning challenging behaviors (such self-isolation) was encouraging and practical	According to calls for more expeditious, practical health research techniques, quick optimization techniques of this kind may be utilized in the future to enhance the speed and efficiency of adaption, refinement, and implementation of interventions
Ross et al. (67)	United Kingdom implemented intervention (HeLP-Diabetes)	Give an illustration of how to create a theoretically based implementation strategy and how to openly disclose it	For the purpose of integrating HeLP-Diabetes into everyday practice, a new implementation strategy was created. The normalization process theory served as a guide for the selection and development of specific component techniques. These tactics included involving local opinion leaders, distributing instructional materials, hosting educational visits and meetings, conducting audits, receiving feedback, and reminding people. Barriers that surfaced during deployment were iteratively addressed with additional solutions. Having trouble allocating funds to put the intervention into practice within ordinary treatment was a major barrier	Others who are working on planning and carrying out implementation activities in regular healthcare can benefit from the knowledge gained from this study. The choice of an acceptable theory to direct the process of implementation and the choice of tactics; making sure that adequate attention is paid to planned implementation and a flexible approach that permits responsiveness to developing hurdles
Bevens et al. (68)	Australia a practical overview	Aims to share information and thoughts from public health academics who have taken part in the process of digitally transforming a face-to-face lifestyle management training program	Information on the digital transformation of lifestyle education programs is scarce, and this is especially true for initiatives focused on chronic conditions. Higher education has produced a significant body of work that has experienced fast digital transition. Much can be learned from this area of study. Additionally, academics looking to design, develop, and implement DHIs have access to a well-established area of design approaches and frameworks	Gives a detailed explanation of how the processes of higher education’s digital transformation can be combined with the use of a current development model for DHIs
Patel et al.(56)	USA cross-sectional analysis	It assessed the current healthcare system’s ability to support digital health treatments and looked at the correlates of the system’s epidemiological, socioeconomic, and geographic contexts	The availability of critical personnel was lower than the availability of IT infrastructure for all locations except subcenters. Higher blood pressure, body mass index, and urban residents were associated with better infrastructure for all hospitals except district hospitals	When compared to apex facilities in India, lower and mid-tier healthcare facilities more commonly lack the IT infrastructure needed to facilitate digital health initiatives. Physical infrastructure gaps were typically higher than staffing ones, indicating that, in addition to IT infrastructure, shortages of key personnel place serious restrictions on the adoption of digital health solutions
Schroeer et al.(58)	Germany a scoping review	Seeks to map the body of research on digital platforms that encourage community meeting in the field of health promotion and prevention	There were two studies on interaction with peers, five studies that used qualitative participatory research, one study on empowerment, and five studies that used crowdsourcing. The digital tools employed ranged greatly and included social networking sites, message boards, websites for online forums, and specialized web hosts and applications. The majority of research cited convenience, flexibility, and anonymity as advantages of digital interventions. Some articles noted drawbacks, such as issues with interpreting data that can only be read in writing or the potential for selection bias brought on by the digital divide	There is a study gap on this subject, as the review only found a few studies that were pertinent to our goal. It was discovered that digital formats are especially well suited for activities where confidentiality and adaptability are advantageous, like online peer-to-peer assistance programs
Harte et al. (46)	USA exploratory	Explains the purpose and plan of a trial that examines the combined impact of community health worker and digital health support on hemoglobin and glucose self-monitoring	The population of interest was low-income people, the study purpose was explicitly to advance knowledge beneficial for increasing health equity, and the study protocols were developed in partnership with frontline community health professionals	It enhances understanding of whether integrating community health worker interventions with digital health can enhance glucose self-monitoring and outcomes related to diabetes in a high-risk group
Chen et al. (69)	China public surveys	Analyze the circumstances and important players in China’s quick deployment of digital health solutions in response to COVID-19, and record and disseminate the lessons collected	The wide adoption of digital health technology revealed contextual elements and important enabling mechanisms in case studies that were identified under each category	The prosperous digital health expanse before COVID-19, the public sector’s flexibility in introducing regulatory flexibilities, and incentives to energize the private sector are among the contextual factors and key permitting mechanisms through the practice of policy instruments to encourage DHIs for COVID-19 in China. These factors also include the route of policy advices affecting the private sector using a regionalized approach
Batta and Iwokwagh (70)	Nigeria inductive content analysis	It examines how Nigerian teaching hospitals make use of social media and new media. It examines whether new and social media are used as public relations tools (to increase their visibility, promote their services, and enhance their corporate image), educational tools (to provide health information, revelation, and education in order to prevent disease and promote health), and social tools (to facilitate communication between people) (to deepen interactions and exchanges between healthcare providers and healthcare recipients)	Nigerian teaching hospitals mostly use new and social media to solicit customer input (100%), provide their vision and mission statements (65%), post details about their administrative and staff structures (65%), and provide contract information (60%). For financial transactions (10%) and the promotion of health (25%), these media are seldom ever used	Teaching hospitals should make more use of social media and new media to give patients and family members a platform to share their stories and to give informed advice on medical and health issues

## Results

The following research question was developed:

What implementing characteristics, concepts and recommendations that encourage digital intervention in public health? The terms “digital health intervention” were recognized as the use of digital, mobile, and wireless technologies to support the achievement of health objectives (71), encompassing both mHealth and eHealth. Arksey and O’Malley (61) advise using a broad definitional approach and propose that search words can be modified and reduced later to manage bibliographic references after the entire breadth of information within a given field has been attained. Given that it applies a uniform analytical framework to all studies, which is considered as a standard practice in scoping reviews, this methodology reflects a “descriptive-analytical” approach to charting.

From the publications, this study obtained both qualitative and quantitative data. This study’s major objective was to conceptually clarify the characteristics of digital intervention for public health.

The results of this study may have also been exaggerated by other search parameters, such as restricting results to English-language articles. The current study’s goal was to regulate the existing status of digital intervention for public health and make recommendations. The methodology was suitable for a policy analysis topic like this one. The limitations found in the literature highlight the need for public health practice information and more rigorous study approaches.

### Concepts of digital intervention for public health in the context of the reviewed articles

The articles focused on diverse concepts for the digital intervention for public health and also on different methods on the topic. Article focus on commercial corporations to adopt cutting-edge technologies (62), advance information and health literacy (63), framework for evaluation that integrates biological and digital methods (7), multidimensional, global public based on an awareness of structural, societal, and individual extents of violence against women and girls (10), factors that influence recruitment and involvement in DHIs (64), DHI’s impact on rural communities’ (66), range of technological developments for the global public health (65), collection of iterative techniques (41), theoretically based implementation strategy (67), share information and thoughts from public health academics, healthcare system’s ability to support digital health treatments (68), encourage community engagement (56), digital health support on hemoglobin (46), circumstances and important players in China’s quick deployment of digital health solutions (69) and teaching hospitals that make use of social media (70).

## Discussion

This scoping review only discovered uncommon studies that used a digital platform to empower substantial community involvement in health promotion and prevention, highlighting a research gap in this area. Digital formats were discovered to be appropriate for situations where obscurity is advantageous. This was evident in the included studies’ qualitative participatory research investigations, notably in the virtual focus groups where contributors had to discuss difficult topics. Additionally, it indicated that anonymity and ease of access were helpful in assisting marginalized and disadvantaged communities, such as through interaction with peers and social exchange programs (58).

With the help of this scoping study, we were able to map the body of research on digital platforms that encourage community involvement in the field of health promotion and prevention. In addition, we obtained a deeper awareness of the fundamental ideas in this field in terms of the sorts of involvement that can be facilitated, the ways to use digital forms, and the advantages and drawbacks associated with them (58).

DHIs are provided through digital channels, such as websites and mobile applications, with the goal of providing care or promoting health (5). Such DHIs are anticipated to combine the effectiveness of individualized therapies with the influence of large-scale population campaigns. DHIs are also meant to expand access and capacity for public health efforts by offering services in places where face-to-face choices are absent or inadequate to satisfy demand (7).

However, it is also important to analyze how institutional inequality, particularly epistemic injustice, affects the content and purposes of the DHIs utilized in public health treatments. They outline and emphasize the significance of violence against women and girls as a global public health concern and briefly evaluate its multifaceted character on structural, societal, and personal levels (10). According to the author, technical solutions like DHIs may be a useful tool in the battle against violence against women or gender inequality, but they must be placed within the larger context of public health that recognizes and the structural components of such fierceness.

The vast amounts of personal information gathered are highly illuminating, and the means for using that information to target digital communications are strong and easily accessible. It is simple to comprehend why academics and professionals in public health are keen to investigate the potential good they may do with them. Such technologies run the risk of discriminatory message targeting against disadvantaged groups. Targeted digital public health interventions pose a risk of interfering with our autonomy by influencing our decision-making. Each situation should be evaluated individually to determine whether the advantages of these interventions exceed the disadvantages. Practitioners should weigh the seriousness of the health risks they are targeting (e.g., promoting a healthy diet as opposed to intervening in suicide cases or eradicating health misinformation during a pandemic) as well as their ability to lessen probable harms (e.g., whether messaging can be clear and collected data respect entities’ privacy) (63).

Collecting the quantitative and qualitative results will produce a strong set of data that can be used to adapt the intervention’s execution with access to the digital health platform as well as to evaluate the DHI. The study illustrates a participatory and community-based component that has the opportunity to have an improved, context-specific influence on local communities’ digital health education by leveraging upon conclusions from both research techniques to enhance the intervention (65).

In addition to a list of the hurdles and implementors that patients and the general public encounter while appealing with and enrolling in DHIs, this review gives an overview of reported engagement and recruitment tactics. In line with the findings of our review, literacy abilities (72) and financial resources (73) do have an impact on people’s capacity to interact with and use DHIs.

Digital technologies must be integrated into the current public healthcare systems since they cannot function alone (74). For instance, as one of many approaches, South Korea and Singapore effectively implemented contact-tracing DHIs to support massive teams of manual contact tracers (66). The digital infrastructure and public health systems’ readiness, which include secondary, primary, and social care systems, will be key factors in the analysis and utilization of these data. With multiple symptom-reporting sites in a single nation, coordination of therapies is especially difficult and runs the danger of fragmentation (66).

The intervention, however, was clinically correct since tight collaboration with clinical stakeholders guaranteed that the information concerning transmission and exposure was compatible with the available data, for instance. Contributors to patient and public involvement (PPI) identified crucial justifications to the intervention’s content, such as whether epidemics can spread through the air as well as surfaces and made sure that evidence about challenging behaviors (like self-isolation) was encouraging and practical (41).

Furthermore, the author has created knowledge about some of the enablers and barriers to putting DHIs into reality. In a system with limited resources, we discovered that requiring personnel to assist patients in registering to use a DHI was a barrier (67). A live intervention’s effectiveness was improved quickly and iteratively to keep pace with the terrifying and continuously changing environment of an international crisis. A rich approach for swift stakeholder assignation was crucial for apprising decisions about how to discourse these obstacles, and the variety of methods assisted in developing a thorough grasp of the potential hurdles to the target behaviors (41).

## Conclusion

Understanding the variables connected to digital interventions for public health begins with this scoping review of the literature. The review has given ideas about the factors that contribute to success and insight into some of the techniques used to identify high achievers, but it has also highlighted the need for new approaches to understanding what counts as high impact and how to enhance elements that are crucial to population health. As, the public health is likely to become more and more digital in the future, the author examines the requirement for the synchronization of global approaches for the regulation, assessment and use of digital technologies in order to improve population health supervision and imminent alertness for diseases.

The author contends that elements that go beyond the inter-individual level must be considered for any intervention technique to be successful and long-lasting. There is little research on the function and importance of DHIs as tactics for addressing the structural and epistemological components. The participants and those around them will gain more awareness about health issues by receiving health messages in a digital format, which may change how they seek out health care. More work is required to develop effective engagement tactics, significantly greater, individualized digital solutions, and to obtain clinical accreditation and support where necessary.

The choice of an appropriate theory to direct the course of implementation and strategy selection is essential. The reporting of implementation strategies using terms that are clear and defined, and using a flexible approach are all important considerations. In addition, physical infrastructure gaps were typically indicating that beyond information technology infrastructure, shortages of indispensable staff enforce significant barriers to the adoption of DHIs.

To sum up, the author’s work outlines an iterative, cross-disciplinary, participatory progression for creating, implementing, and appraising DHI, emphasizing the adjacent collaboration between behavior scientists, designers, data engineers, software developers, and data scientists as well as on a constant reaction circle from end users. A defined approach for swift stakeholder involvement was crucial for guiding decisions about how to discourse these obstacles, and the variety of ways contributed to the development of a deep consideration of the potential barriers to the target behaviors. Making sure the intervention’s content is inspiring, reliable, and convincing may be more crucial for fostering engagement than making changes to the intervention’s design (66).

DHI offers a viewpoint that emphasizes a considerable larger series of issues related to the sociotechnical system involved by a specific digital health technology and the health of the numerous communities. This study could be used in other areas of public health policy and practice and will attend as a source for enduring discussion in this area.

## Author contributions

HA held the main parts of the research, writing, collecting the data, and results and discussion. AA helped HA in reviewing the paper and gave notes. All authors contributed to the article and approved the submitted version.

## Conflict of interest

The authors declare that the research was conducted in the absence of any commercial or financial relationships that could be construed as a potential conflict of interest.

## Publisher’s note

All claims expressed in this article are solely those of the authors and do not necessarily represent those of their affiliated organizations, or those of the publisher, the editors and the reviewers. Any product that may be evaluated in this article, or claim that may be made by its manufacturer, is not guaranteed or endorsed by the publisher.

## Abbreviations

DHI: Digital Health Intervention
DHIs: Digital Health Interventions
PPI: Patient and Public Involvement

## References

[1] Chou WYS Oh A Klein WM. Addressing health-related misinformation on social media. JAMA. (2018) 320:2417–8. doi: 10.1001/jama.2018.16865, PMID: 30428002

[2] Human Rights Watch (2020). Myanmar: end world’s longest internet shutdown. Available at: https://www.hrw.org/news/2020/06/19/myanmar-end-worlds-longestinternetshutdown.

[3] Rimmer A. COVID-19: disproportionate impact on ethnic minority healthcare workers will be explored by government. Br Med J. (2020) 369:m1562. doi: 10.1136/bmj.m1562 32303494

[4] World Health Organization. Education for health: a manual on health education in primary health care World Health Organization (1988) Available at: https://apps.who.int/iris/handle/10665/77769.

[5] Murray E Hekler EB Andersson G Collins LM Doherty A Hollis C . Evaluating digital health interventions: key questions and approaches. Am J Prev Med. (2016) 51:843–51. doi: 10.1016/j.amepre.2016.06.008, PMID: 27745684 PMC5324832

[6] Tognotti E. Lessons from the history of quarantine, from plague to influenza A. Emerg Infect Dis. (2013) 19:254. doi: 10.3201/eid1902.120312, PMID: 23343512 PMC3559034

[7] Karpathakis K Libow G Potts HW Dixon S Greaves F Murray E. An evaluation service for digital public health interventions: user-centered design approach. J Med Internet Res. (2021) 23:e28356. doi: 10.2196/28356, PMID: 34494965 PMC8459216

[8] Blandford A Gibbs J Newhouse N Perski O Singh A Murray E. Seven lessons for interdisciplinary research on interactive digital health interventions. Digit Health. (2018) 4:2055207618770325. doi: 10.1177/2055207618770325, PMID: 29942629 PMC6016567

[9] Coghlan S D’Alfonso S. Digital phenotyping: an epistemic and methodological analysis. Philos Technol. (2021) 34:1905–28. doi: 10.1007/s13347-021-00492-1, PMID: 34786325 PMC8581123

[10] Sauerborn E Eisenhut K Ganguli-Mitra A Wild V. Digitally supported public health interventions through the lens of structural injustice: the case of mobile apps responding to violence against women and girls. Bioethics. (2022) 36:71–6. doi: 10.1111/bioe.12965, PMID: 34668600

[11] Oudshoorn N. Telecare technologies and the transformation of healthcare. Houndmills, UK: Palgrave Macmillan. (2011).

[12] Greenhalgh T Wood GW Bratan T Stramer K Hinder S. Patients’ attitudes to the summary care record and HealthSpace: qualitative study. BMJ. (2008) 336:1290–5. doi: 10.1136/bmj.a114, PMID: 18511764 PMC2413413

[13] Bardus M Blake H Lloyd S Suzanne Suggs L. Reasons for participating and not participating in a e-health workplace physical activity intervention: A qualitative analysis. Int. J. Workplace Health Manag. (2014) 7:229–46.

[14] Lorimer K Martin S McDaid LM. The views of general practitioners and practice nurses towards the barriers and facilitators of proactive, internet-based chlamydia screening for reaching young heterosexual men. BMC Fam Pract. (2014) 15:1–10. doi: 10.1186/1471-2296-15-127 24972919 PMC4096584

[15] Trujillo Gómez JM Díaz-Gete L Martín-Cantera C Fábregas Escurriola M Lozano Moreno M Burón Leandro R . Intervention for smokers through new communication technologies: what perceptions do patients and healthcare professionals have? A qualitative study. PLoS One. (2015) 10:e0137415. doi: 10.1371/journal.pone.0137415, PMID: 26340346 PMC4560416

[16] Winkelman WJ Leonard KJ Rossos PG. Patient-perceived usefulness of online electronic medical records: employing grounded theory in the development of information and communication technologies for use by patients living with chronic illness. J Am Med Inform Assoc. (2005) 12:306–14. doi: 10.1197/jamia.M1712, PMID: 15684128 PMC1090462

[17] Lundgren J Johansson P Jaarsma T Andersson G Köhler AK. Patient experiences of web-based cognitive behavioral therapy for heart failure and depression: qualitative study. J Med Internet Res. (2018) 20:e10302. doi: 10.2196/10302, PMID: 30185405 PMC6231888

[18] Wilhelmsen M Lillevoll K Risør MB Høifødt R Johansen ML Eisemann M . Motivation to persist with internet-based cognitive behavioural treatment using blended care: a qualitative study. BMC Psychiatry. (2013) 13:1–9. doi: 10.1186/1471-244X-13-296 24199672 PMC4226213

[19] Wachtler C Coe A Davidson S Fletcher S Mendoza A Sterling L . Development of a mobile clinical prediction tool to estimate future depression severity and guide treatment in primary care: user-centered design. JMIR Mhealth Uhealth. (2018) 6:e9502. doi: 10.2196/mhealth.9502, PMID: 29685864 PMC5938570

[20] Pagliari C Burton C McKinstry B Szentatotai A David D Ferrini L . Psychosocial implications of avatar use in supporting therapy for depression. Annu Rev Cyberther Telemed. (2012) 2012:329–333. doi: 10.1111/nyas.13336 22954882

[21] Gonsalves PP Hodgson ES Kumar A Aurora T Chandak Y Sharma R . Design and development of the “POD adventures” smartphone game: a blended problem-solving intervention for adolescent mental health in India. Front Public Health. (2019) 7:238. doi: 10.3389/fpubh.2019.00238 31508404 PMC6716539

[22] Povey J Mills PPJR Dingwall KM Lowell A Singer J Rotumah D . Acceptability of mental health apps for aboriginal and Torres Strait islander Australians: a qualitative study. J Med Internet Res. (2016) 18:e5314. doi: 10.2196/jmir.5314, PMID: 26969043 PMC4825593

[23] Hunter-Jones JJ Gilliam SM Carswell AL Hansen NB. Assessing the acceptability of a mindfulness-based cognitive therapy intervention for African-American women living with HIV/AIDS. J Racial Ethn Health Disparities. (2019) 6:1157–66. doi: 10.1007/s40615-019-00617-5, PMID: 31332688

[24] Henshall C Marzano L Smith K Attenburrow MJ Puntis S Zlodre J . A web-based clinical decision tool to support treatment decision-making in psychiatry: a pilot focus group study with clinicians, patients and carers. BMC Psychiatry. (2017) 17:1–10. doi: 10.1186/s12888-017-1406-z 28732477 PMC5521138

[25] Walsh DM Moran K Cornelissen V Buys R Cornelis N Woods C. Electronic health physical activity behavior change intervention to self-manage cardiovascular disease: qualitative exploration of patient and health professional requirements. JMIR. (2018) 20:e163. 29739740 10.2196/jmir.9181 PMC11340777

[26] Feijt MA de Kort YA Bongers IM WA IJ. Perceived drivers and barriers to the adoption of eMental health by psychologists: the construction of the levels of adoption of eMental health model. J Med Internet Res. (2018) 20:e9485. doi: 10.2196/jmir.9485, PMID: 29691215 PMC5941096

[27] Apolinário-Hagen J Vehreschild V Alkoudmani RM. Current views and perspectives on e-mental health: an exploratory survey study for understanding public attitudes toward internet-based psychotherapy in Germany. JMIR Ment health. (2017) 4:e6375. doi: 10.2196/mental.6375, PMID: 28232298 PMC5378055

[28] Jordan SE Shearer EM. An exploration of supervision delivered via clinical video telehealth (CVT). Train Educ Prof Psychol. (2019) 13:323. doi: 10.1037/tep0000245, PMID: 30261806

[29] Kumarasiri J Jubb C. Framing of climate change impacts and use of management accounting practices. Asian Acad Manag J Account Finance. (2017) 13. doi: 10.21315/aamjaf2017.13.2.3

[30] Huerta-Ramos E Escobar-Villegas MS Rubinstein K Unoka ZS Grasa E Hospedales M . Measuring users’ receptivity toward an integral intervention model based on mHealth solutions for patients with treatment-resistant schizophrenia (m-RESIST): A qualitative study. JMIR Mhealth Uhealth. (2016) 4:e5716. doi: 10.2196/mhealth.5716, PMID: 27682896 PMC5062002

[31] Donkin L Glozier N. Motivators and motivations to persist with online psychological interventions: a qualitative study of treatment completers. J Med Internet Res. (2012) 14:e2100. doi: 10.2196/jmir.2100, PMID: 22743581 PMC3414905

[32] Wallin E Norlund F Olsson EMG Burell G Held C Carlsson T. Treatment activity, user satisfaction, and experienced usability of internet-based cognitive behavioral therapy for adults with depression and anxiety after a myocardial infarction: mixed-methods study. J Med Internet Res. (2018) 20:e9690. doi: 10.2196/jmir.9690, PMID: 29549067 PMC5878371

[33] Anderson AP Fellows AM Binsted KA Hegel MT Buckey JC. Autonomous, computer-based behavioral health countermeasure evaluation at HI-SEAS Mars analog. Aerosp Med Hum Perform. (2016) 87:912–20. doi: 10.3357/AMHP.4676.2016, PMID: 27779949

[34] Jonathan G Carpenter-Song EA Brian RM Ben-Zeev D. Life with FOCUS: a qualitative evaluation of the impact of a smartphone intervention on people with serious mental illness. Psychiatr Rehabil J. (2019) 42:182. doi: 10.1037/prj0000337, PMID: 30589278 PMC10438016

[35] Williams A Fossey E Farhall J Foley F Thomas N. Recovery after psychosis: qualitative study of service user experiences of lived experience videos on a recovery-oriented website. JMIR Ment Health. (2018) 5:e9934. doi: 10.2196/mental.9934, PMID: 29739737 PMC5964305

[36] Görges F Oehler C von Hirschhausen E Hegerl U Rummel-Kluge C. GET. HAPPY2—user perspectives on an internet-based self-management positive psychology intervention among persons with and without depression: results from a retrospective survey. J Clin Psychol. (2020) 76:1030–46. doi: 10.1002/jclp.22886, PMID: 31714609

[37] Lawal AM Idemudia ES Senyatsi T. Emotional intelligence and mental health: an exploratory study with south African university students. J Psychol Afr. (2018) 28:492–7. doi: 10.1080/14330237.2018.1540229

[38] Eisner E Drake RJ Berry N Barrowclough C Emsley R Machin M . Development and long-term acceptability of ExPRESS, a mobile phone app to monitor basic symptoms and early signs of psychosis relapse. JMIR Mhealth Uhealth. (2019) 7:e11568. doi: 10.2196/11568, PMID: 30924789 PMC6460313

[39] Edbrooke-Childs J Edridge C Averill P Delane L Hollis C Craven MP . A feasibility trial of power up: smartphone app to support patient activation and shared decision making for mental health in young people. JMIR Mhealth Uhealth. (2019) 7:e11677. doi: 10.2196/11677, PMID: 31165709 PMC6682268

[40] Borghouts J Eikey E Mark G De Leon C Schueller SM Schneider M . Barriers to and facilitators of user engagement with digital mental health interventions: systematic review. J Med Internet Res. (2021) 23:e24387. doi: 10.2196/24387, PMID: 33759801 PMC8074985

[41] Morton K Ainsworth B Miller S Rice C Bostock J Denison-Day J . Adapting behavioral interventions for a changing public health context: a worked example of implementing a digital intervention during a global pandemic using rapid optimisation methods. Front Public Health. (2021) 9:668197. doi: 10.3389/fpubh.2021.668197, PMID: 33981669 PMC8109268

[42] McCrabb S Mooney K Elton B Grady A Yoong SL Wolfenden L. How to optimise public health interventions: a scoping review of guidance from optimisation process frameworks. BMC Public Health. (2020) 20:1–12. doi: 10.1186/s12889-020-09950-5 33267844 PMC7709329

[43] Long JA Jahnle EC Richardson DM Loewenstein G Volpp KG. Peer mentoring and financial incentives to improve glucose control in African American veterans: a randomized trial. Ann Intern Med. (2012) 156:416–24. doi: 10.7326/0003-4819-156-6-201203200-00004, PMID: 22431674 PMC3475415

[44] Palmas W March D Darakjy S Findley SE Teresi J Carrasquillo O . Community health worker interventions to improve glycemic control in people with diabetes: a systematic review and meta-analysis. J Gen Intern Med. (2015) 30:1004–12. doi: 10.1007/s11606-015-3247-0, PMID: 25735938 PMC4471021

[45] Heisler M Vijan S Makki F Piette JD. Diabetes control with reciprocal peer support versus nurse care management: a randomized trial. Ann Intern Med. (2010) 153:507–15. doi: 10.7326/0003-4819-153-8-201010190-00007, PMID: 20956707 PMC4117390

[46] Harte R Norton L Whitehouse C Lorincz I Jones D Gerald N . Design of a randomized controlled trial of digital health and community health worker support for diabetes management among low-income patients. Contemp Clin Trials Commun. (2022) 25:100878. doi: 10.1016/j.conctc.2021.100878, PMID: 34977421 PMC8688867

[47] O’Cathain A Croot L Duncan E Rousseau N Sworn K Turner KM . Guidance on how to develop complex interventions to improve health and healthcare. BMJ Open. (2019) 9:e029954. doi: 10.1136/bmjopen-2019-029954, PMID: 31420394 PMC6701588

[48] Chambers DA. Considering the intersection between implementation science and COVID-19. Implement Res Pract. (2020) 1:0020764020925994. doi: 10.1177/0020764020925994, PMID: 36915854 PMC9981883

[49] Yousefi Nooraie R Shelton RC Fiscella K Kwan BM McMahon JM. The pragmatic, rapid, and iterative dissemination and implementation (PRIDI) cycle: adapting to the dynamic nature of public health emergencies (and beyond). Health Res Policy Syst. (2021) 19:1–10. doi: 10.1186/s12961-021-00764-4 34348732 PMC8335455

[50] Bereczky T. Patient advocacy-with a feeling patient citizenship-the description of an affective model of patient advocacy In: Doctoral dissertation. Hungary: ELTE University (2019) Available at: https://www.academia.edu/42773922/Patient_Advocacy_With_a_Feeling_Patient_citizenship_The_description_of_an_affective_model_of_patient_advocacy

[51] Biddle MS Gibson A Evans D. Attitudes and approaches to patient and public involvement across Europe: a systematic review. Health Soc Care Community. (2021) 29:18–27. doi: 10.1111/hsc.13111, PMID: 32705752

[52] Huckvale K Venkatesh S Christensen H. Toward clinical digital phenotyping: a timely opportunity to consider purpose, quality, and safety. NPJ Digi Med. (2019) 2:1–11. doi: 10.1038/s41746-019-0166-1 PMC6731256 31508498

[53] Algeo N Hunter D Cahill A Dickson C Adams J. Usability of a digital self-management website for people with osteoarthritis: a UK patient and public involvement study. Int J Ther Rehabil. (2017) 24:78–82. doi: 10.12968/ijtr.2017.24.2.78

[54] Aguayo GA Goetzinger C Scibilia R Fischer A Seuring T Tran VT . Methods to generate innovative research ideas and improve patient and public involvement in modern epidemiological research: review, patient viewpoint, and guidelines for implementation of a digital cohort study. J Med Internet Res. (2021) 23:e25743. doi: 10.2196/25743, PMID: 34941554 PMC8738987

[55] Pfadenhauer LM Gerhardus A Mozygemba K Lysdahl KB Booth A Hofmann B . Making sense of complexity in context and implementation: the context and implementation of complex interventions (CICI) framework. Implement Sci. (2017) 12:1–17. doi: 10.1186/s13012-017-0552-5 28202031 PMC5312531

[56] Patel SA Vashist K Jarhyan P Sharma H Gupta P Jindal D . A model for national assessment of barriers for implementing digital technology interventions to improve hypertension management in the public health care system in India. BMC Health Serv Res. (2021) 21:1–11. doi: 10.1186/s12913-021-06999-9 34654431 PMC8517936

[57] Nared J Bole D. Participatory research and planning in practice. Berlin, Germany: Springer. (2020). 227 p.

[58] Schroeer C Voss S Jung-Sievers C Coenen M. Digital formats for community participation in health promotion and prevention activities: a scoping review. Front Public Health. (2021) 9:713159. doi: 10.3389/fpubh.2021.713159, PMID: 34869143 PMC8634959

[59] Zanaboni P Ngangue P Mbemba GIC Schopf TR Bergmo TS Gagnon MP. Methods to evaluate the effects of internet-based digital health interventions for citizens: systematic review of reviews. J Med Internet Res. (2018) 20:e10202. doi: 10.2196/10202, PMID: 29880470 PMC6013714

[60] Mays N Roberts E Popay J. Synthesising research evidence In: Fulop N Allen P Clarke A Black N, editors. Studying the organization and delivery of health services: research methods. London: Routledge. (2001)

[61] Arksey H O'Malley L. Scoping studies: towards a methodological framework. Int J Soc Res Methodol. (2005) 8:19–32. doi: 10.1080/1364557032000119616, PMID: 37256179

[62] Burke C Bloss C. Social media surveillance in schools: rethinking public health interventions in the digital age. J Med Internet Res. (2020) 22:e22612. doi: 10.2196/22612, PMID: 33179599 PMC7691090

[63] Susser D. Ethical considerations for digitally targeted public health interventions. Am J Public Health. (2020) 110:S290–1. doi: 10.2105/AJPH.2020.305758, PMID: 33001734 PMC7532326

[64] O’Connor S Hanlon P O’Donnell CA Garcia S Glanville J Mair FS. Understanding factors affecting patient and public engagement and recruitment to digital health interventions: a systematic review of qualitative studies. BMC Med Inform Decis Mak. (2016) 16:1–15. doi: 10.1186/s12911-016-0359-3 27630020 PMC5024516

[65] Holst C Sukums F Ngowi B Diep LM Kebede TA Noll J . Digital health intervention to increase health knowledge related to diseases of high public health concern in Iringa, Tanzania: protocol for a mixed methods study. JMIR Res Protoc. (2021) 10:e25128. doi: 10.2196/25128, PMID: 33885369 PMC8103301

[66] Budd J Miller BS Manning EM Lampos V Zhuang M Edelstein M . Digital technologies in the public-health response to COVID-19. Nat Med. (2020) 26:1183–92. doi: 10.1038/s41591-020-1011-4, PMID: 32770165

[67] Ross J Stevenson F Dack C Pal K May C Michie S . Developing an implementation strategy for a digital health intervention: an example in routine healthcare. BMC Health Serv Res. (2018) 18:1–13. doi: 10.1186/s12913-018-3615-7 30340639 PMC6194634

[68] Merolli M. Insights from public health researchers into the digital transformation of an educational lifestyle course. Healthier Lives, Digitally Enabled: Selected Papers from the Digital Health Institute Summit 2020, vol. 276. (2021) p. 14.

[69] Chen M Xu S Husain L Galea G. Digital health interventions for COVID-19 in China: a retrospective analysis. Intell Med. (2021) 1:29–36. doi: 10.1016/j.imed.2021.03.001 34447602 PMC8079943

[70] Batta HE Iwokwagh NS. Optimising the digital age health-wise: utilisation of new/social media by Nigerian teaching hospitals. Procedia Soc Behav Sci. (2015) 176:175–85. doi: 10.1016/j.sbspro.2015.01.459

[71] World Health Organization. Monitoring and evaluating digital health interventions: a practical guide to conducting research and assessment. Geneva, Switzerland: World Health Organization (2016).

[72] Kontos E Blake KD Chou WYS Prestin A. Predictors of eHealth usage: insights on the digital divide from the health information National Trends Survey 2012. J Med Internet Res. (2014) 16:e3117. doi: 10.2196/jmir.3117, PMID: 25048379 PMC4129114

[73] Neter E Brainin E. eHealth literacy: extending the digital divide to the realm of health information. J Med Internet Res. (2012) 14:e1619. doi: 10.2196/jmir.1619, PMID: 22357448 PMC3374546

[74] Gong M Liu L Sun X Yang Y Wang S Zhu H. Cloud-based system for effective surveillance and control of COVID-19: useful experiences from Hubei, China. J Med Int Res. (2020) 22:e18948. doi: 10.2196/18948, PMID: 32287040 PMC7179239

